# Quality control of *Ganoderma lucidum* by using C, H, O, and N stable isotopes and C and N contents for geographical traceability

**DOI:** 10.3389/fpls.2023.1234729

**Published:** 2023-10-11

**Authors:** Ying Zhang, Kunxia Jiang, Sisi Chen, Lina Wang, Xun Zhang, Wen Xu, Mun Fei Yam, Changhui Wu, Wei Xu, Yu Lin

**Affiliations:** ^1^ College of Pharmacy, Fujian University of Traditional Chinese Medicine, Fuzhou, Fujian, China; ^2^ Centre of Biomedical Research & Diversity of Development, Fujian University Traditional Chinese Medicine, Fuzhou, Fujian, China; ^3^ Innovation and Transformation Center of Science and Technology, Fujian University of Traditional Chinese Medicine, Fuzhou, Fujian, China; ^4^ Department of Pharmacology, School of Pharmaceutical Sciences, University Sains Malaysia, Minden, Penang, Malaysia; ^5^ Research and Development Department, Fujian Xianzhilou Biological Science & Technology Co., Ltd., Fuzhou, China

**Keywords:** *Ganoderma lucidum*, stable isotope ratio, chemometrics, growth stages, geographical origin

## Abstract

**Rationale:**

*Ganoderma lucidum* (*G. lucidum*) is a popular medicinal fungus that has been used in traditional medicine for decades, with its provenance influencing its medicinal and commercial worth. The amount of active ingredients and the price of *G. lucidum* from different origins vary significantly; hence, fraudulent labeling is common. Reliable techniques for *G. lucidum* geographic verification are urgently required to safeguard the interests of consumers, producers, and honest dealers. A stable isotope is widely acknowledged as a useful traceability technique and could be developed to confirm the geographical origin of *G. lucidum*.

**Methods:**

*G. lucidum* samples from various sources and in varying stages were identified by using *δ* ^13^C, *δ*D, *δ* ^18^O, *δ* ^15^N, C, and N contents combined with chemometric tools. Chemometric approaches, including PCA, OPLS-DA, PLS, and FLDA models, were applied to the obtained data. The established models were used to trace the origin of *G. lucidum* from various sources or track various stages of *G. lucidum*.

**Results:**

In the stage model, the *δ* ^13^C, *δ*D, *δ* ^18^O, *δ* ^15^N, C, and N contents were considered meaningful variables to identify various stages of *G. lucidum* (bud development, growth, and maturing) using PCA and OPLS-DA and the findings were validated by the PLS model rather than by only four variables (*δ* ^13^C, *δ*D, *δ* ^18^O, and *δ* ^15^N). In the origin model, only four variables, namely *δ* ^13^C, *δ*D, *δ* ^18^O, and *δ* ^15^N, were used. PCA divided *G. lucidum* samples into four clusters: A (Zhejiang), B (Anhui), C (Jilin), and D (Fujian). The OPLS-DA model could be used to classify the origin of *G. lucidum*. The model was validated by other test samples (*Pseudostellaria heterophylla*), and the external test (*G. lucidum*) by PLS and FLDA models demonstrated external verification accuracy of up to 100%.

**Conclusion:**

C, H, O, and N stable isotopes and C and N contents combined with chemometric techniques demonstrated considerable potential in the geographic authentication of *G. lucidum*, providing a promising method to identify stages of *G. lucidum*.

## 1 Introduction

*G. lucidum*, a traditional medicinal mushroom that has been used for several millennia to treat various common ailments, is one of the best-known medicinal macrofungi in the world. The pharmacological activities of *G. lucidum* are widely recognized and have been used for centuries to promote health and longevity. Moreover, *G. lucidum* is listed in the Herbal Medicines of the USP, the Chinese Pharmacopoeia, and the Japanese Pharmacopoeia. China is the main source of the *G. lucidum* industry worldwide; for example, Fujian *G. lucidum* has been certified as an organic food in the United States, Europe, Japan, and China. *G. lucidum* has recently been used to produce various food and health products due to its potentially pharmacologically active components and healthful features, thereby attaining immense economic value. The perceived nutritional and healthy values of *G. lucidum* have been accepted globally, and its annual economic output is more than US$2.5 billion (Ren et al., 2020).

Certain production regions suitable for *G. lucidum* growth, called “daodi” in China, have specific strains and cultivation practices. Triterpenoids and polysaccharides are the prime bioactive components responsible for the therapeutic activities of *G. lucidum*, which have been reported to be anti-inflammatory and anti-cancer, to decrease obesity, and ameliorate metabolic dysfunctions (Chang et al., 2015; Ahmad, 2020; Sang et al., 2021; Wang et al., 2019). The composition of its chemical components, including triterpenoids, polysaccharides, proteins, alkaloids, and vitamins, which are key sources of its pharmacological active constituents, is affected by its growing conditions. Studies have revealed discrepancies in the protein and trace element content of *G. lucidum* grown in China and Iberia and also differences in the ganoderic acid and triterpenoid content of *G. lucidum* from various sources (Ha et al., 2015; Zhang et al., 2019; Fraile et al., 2021), resulting in an unstable medicinal value and curative effect of extended products. Therefore, the geographical origin is considered to be a key factor affecting the safety and quality of *G. lucidum*. This factor raises concerns regarding the authenticity and traceability of mushroom species and the impact of commercial medicinal plant extracts on the *G. lucidum* extraction and fractionation sector. The capacity to trace the geographical origin of *G. lucidum* will increase its value in traditional Chinese medicine (TCM) and cuisine.

Different stages of *G. lucidum* show different pharmacological activities. Basswood cultivation (one way of log planting) is a certified organic food of *G. lucidum* cultivation in the United States, European Union, Republic of Korea, Japan, and China (Surya et al., 2018). Temperature, humidity, CO_2_ content, and other environmental factors affect the growth of *G. lucidum* during the planting process (Bijalwan et al., 2021), which may lead to changes in stable isotopes or bioactive compounds in different stages of *G. lucidum*. The changes in bioactive compounds may also result in variations in stable isotopes at different stages. Stripes during the spore maturity stage had the highest total phenols and flavonoids, resulting in a high level of antioxidant activity, but bud-developing stage stripes had substantially high levels of ganoderic acid B, C_2_, and G based on previous research (Ren et al., 2020). Several *G. lucidum*-based products are available in the form of nutriceuticals. Some of these products are marketed as dietary supplements and are widely consumed in some countries. Changes in the nutritional content of *G. lucidum* during its growth will undoubtedly result in alterations to its efficacy (Chen et al., 2012). Therefore, developing a technology for distinguishing different stages of *G. lucidum* is necessary to provide a scientific basis for the advancement and utilization of *G. lucidum*.

The authors are aware that numerous chemical strategies based on chromatographic and spectral analysis methodologies have been developed for determining the geographical origin and applied to a range of TCM, such as techniques based on FT-NIR, UHPLC-QqQ-MS/MS, GC-MS, and LC-MS (Chen et al., 2008; Fu et al., 2017; Zhao et al., 2021; Singh et al., 2022). However, they cannot effectively distinguish the geographic origin of TCM due to insufficient compound information. A chemometrics strategy that combines stable isotope ratios has emerged as a promising approach for distinguishing regional origins, particularly in rice, honey, milk, and wolfberry (Magdas et al., 2008; Wang et al., 2020; Zhao et al., 2020; Gong et al., 2022). A stoichiometric strategy incorporating stable isotope ratios has also emerged as a highly promising approach for identifying the geographic origin of various products. This strategy has been successfully applied to high-value food or medical products, such as honey’s authenticity and global geographic origin (Zhou et al., 2018). The application of stable isotopes to the tracing of *G. lucidum*, a valuable traditional Chinese medicinal ingredient, has not been documented in any study.

The current study aims to further investigate the potential of stable isotopes for the geographic authentication of *G. lucidum*. The isotope ratios of *δ* ^13^C, *δ*D, *δ* ^18^O, *δ* ^15^N, C%, and N% in *G. lucidum* samples produced in four provinces of China were measured by isotope ratio mass spectrometry. Chemometric methods, including principal component analysis (PCA), orthogonal partial least squares-discriminant analysis (OPLS-DA), PLS, and Fisher linear discriminant analysis (FLDA), were applied to evaluate the discriminant power of these data.

## 2 Materials and methods

### 2.1 *G. Lucidum* samples

**Figure 1:**
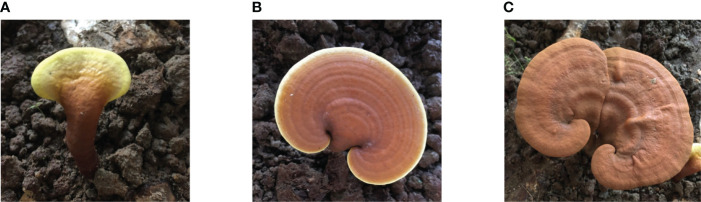
*G*. *lucidum* at different stages. **(A)** Bud-developing stage, **(B)** Growth stage, and **(C)** Maturing stage.

The *G. lucidum* from Fujian was provided by Fujian Xian-Zhi-Lou Technology Co., Ltd. The strain of *G. lucidum* was JB-A119-20191010. It was incubated for 7 days at 28°C on potato dextrose agar (PDA) (medium: potato, 200 g; dextrose, 20 g; agar, 20 g; and deionized water, 1 L). The inoculated substrate was incubated at 28°C for mycelial colonization. The air temperature in the cultivation room was maintained at 28°C, and the relative humidity was set at 70%–80%. The grown mycelium was homogenized after 15–20 days of cultivation. The culture medium was autoclaved at 121°C for 2 h. The fruiting body samples were cultivated by the solid method using wood (the wood of *Castanopsis sclerophylla* used for cultivating *G. lucidum* was taken from local forests). Before planting, the cultivation site had been aired, cleaned, and slightly lime-disinfected and had proper ventilation (CO_2_ 0.1%–0.3%). The experiment was conducted at the three growth stages of bud development, growth, and maturing. Bud development stage: After inoculation, the mycelia of *G. lucidum* began to germinate. Primordium culture conditions were maintained at 28°C–30°C, 90%–95% relative humidity, 0.03%–0.1% CO_2_, and a light intensity of 100 lux. The initial sampling was conducted approximately 35–40 days after inoculation, which is when the bud-developing stage and differentiation had begun. Growth stage: The second sampling was conducted at the growth stage, approximately 55–60 days after inoculation, which is when the primordial grows long and stripes form. The growth stage began after elongation. The color of the caps deepens as they grow, demonstrating a deep center and white edges. The culture conditions of fruiting bodies were maintained at 25°C–28°C, relative humidity at 80%–95%, CO_2_ of 0.03%–0.1%, and a light intensity of 150–200 lux. Maturing stage: The last sampling was performed at the maturing stage approximately 70–80 days after the inoculation. The culture conditions of fruiting bodies were maintained at 25°C–28°C, relative humidity at 80%–95%, CO_2_ at 0.03%–0.05%, and a light intensity of 150–200 lux. *G. lucidum* at different stages, when the white edge of the cap disappears and hardens, is shown in **Figure 1**.

**Figure 2:**
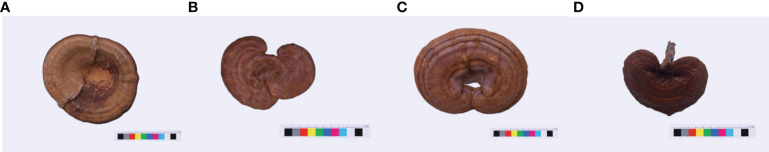
*G*. *lucidum* from different areas. **(A)** Zhejiang (ZJ), **(B)** Anhui (AH), **(C)** Jilin (JL), and **(D)** Fujian (FJ).

**Figure 3:**
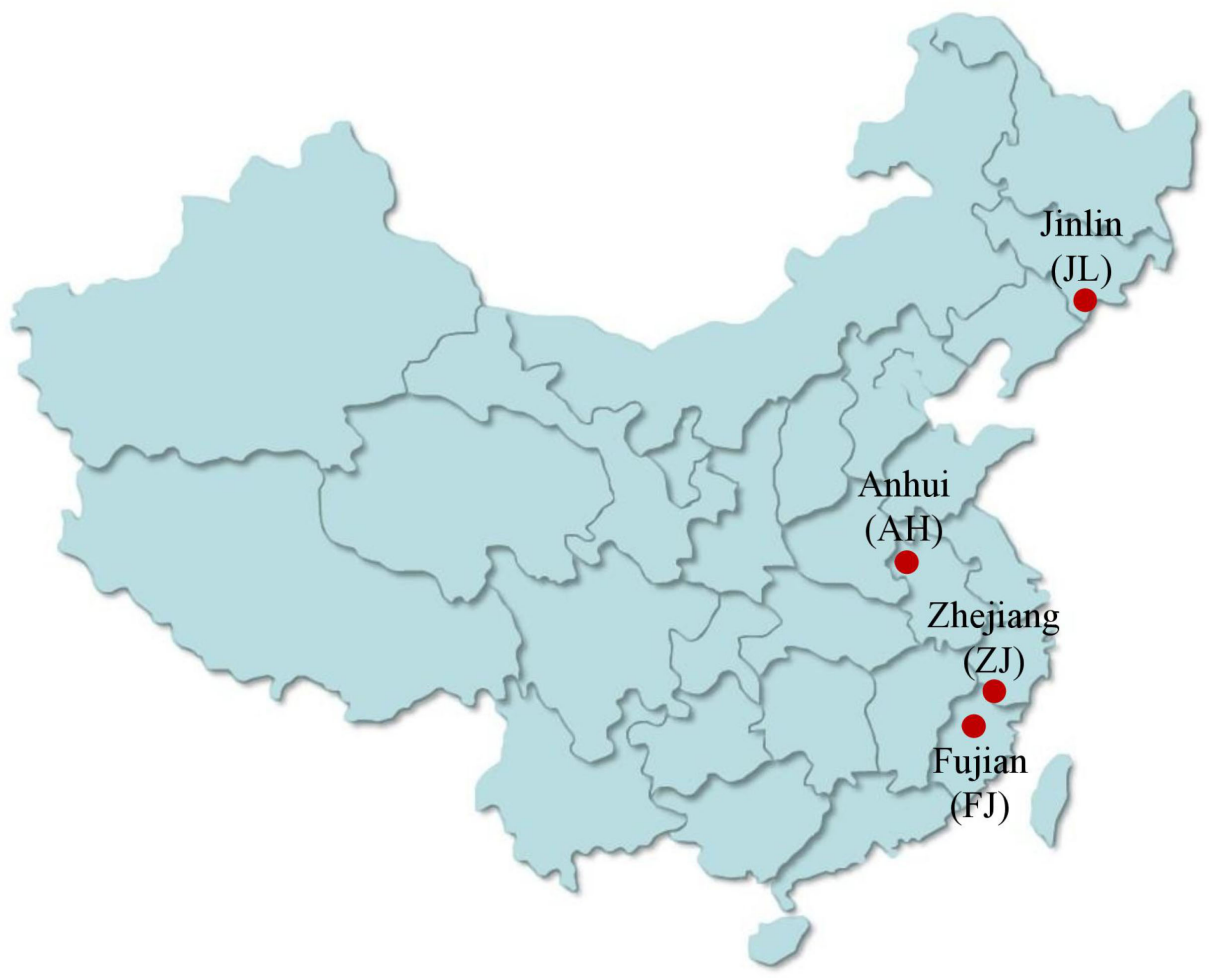
The sample locations of *G. lucidum* in China.

*G. Lucidum* collected from Zhejiang, Anhui, and Jilin was grown on wood logs and purchased from the local “Daodi” market (maturity stage). The origin of *G. lucidum* includes latitude, longitude, and specific parameters of the planting process as shown in **Table 1**. The pictures of *G. lucidum* from different origins and the specific sampling locations are presented in **Figures 2** and **3**, respectively. The *Pseudostellaria heterophylla* samples used for heterogeneous validation were collected from Anhui (*n* = 3) and Fujian (*n* = 3). All the samples were dried at 45°C in a blast drying oven (9240A, Shanghai Jing Hong Laboratory Instrument Co., Ltd.). The dried samples were then ground into a fine powder with a ball mill, sieved through a 100-mesh sieve, and then subjected to stable isotope ratio analysis.

**Table 1:** Geographical origin and environmental conditions of *G. lucidum* samples.

Region	ZJ	AH	JL	FJ
Latitude, °N	27°42′~30°33′	31°01′~35°05′	41°21′~44°40′	26°30′~28°20′
Longitude, °E	118°21′~120°30′	115°20′~117°14′	125°40′~128°18′	117°00′~119°25′
Fruiting body growth Temperature, °C	20~32	20~32	20~30	25~35

ZJ, Zhejiang; AH, Anhui; JL, Jilin; FJ, Fujian.

### 2.2 Stable isotope ratios analysis

The stable isotope ratios (C and N) and the carbon and nitrogen contents were measured with stable isotope mass spectrometry (Element analyzer-isotope ratio mass spectrometers (EA-IRMS), Thermo). The EA-IRMS conditions were set as follows: the reactor temperature was 960°C, and gases produced by the oxidation reactor were changed into He in the reducing furnace. He was then used as a carrier gas at a flow rate of 90 mL·min^−1^. The gases were subsequently separated and measured by EA-IRMS. Reference materials, including IAEA-600 and USGS40, were used for calibration. The sample powders were accurately weighed at 1 mg and then packed in a tin cup for the analysis of EA-IRMS.

The stable isotope ratios (O and H) were measured with elemental analysis isotope ratio mass spectrometry (high-temperature pyrolysis element analyzer and gas isotope ratio mass spectrometer (HT-IRMS), Thermo). The HT-IRMS conditions were set as follows: the reactor temperature was 1,380°C, He was used as carrier gas at a flow rate of 100 mL·min^−1^, and the column temperature was 600°C. Reference materials, including EMA-P1, EMA-P2, and IAEA-601, were used for calibration. The sample powders were accurately weighed at 3 mg and then packed in a tin cup for the analysis of HT-IRMS.

Stability checks of the used reference gases were continuously performed by determining International Atomic Energy Agency standards with defined ^13^C/^12^C, ^15^N/^14^N, ^18^O/^16^O, and D/^1^H ratios. Isotope ratios were expressed in per mil (‰) deviation relative to the V-PDB, V-SMOW, and N_2_ international standards. Values were calculated using the following formula:


$$\delta sample=(\frac{Rsample-Rstandard}{Rstantard}),$$

where *R_sample_* is the ratio of the heaviest to the lightest stable isotope in the sample, and *R_standard_* is the ratio of the heaviest to the lightest stable isotope in the international reference standards, namely, V-PDB, air, and V-SMOW for *δ* ^13^C, *δ* ^15^N, and *δ* ^18^O, and *δ*D values, respectively.

### 2.3 Statistical analysis

The results were presented as mean values ± SD (standard deviation). One-way analysis of variance was utilized for the statistical analysis (ANOVA). The FLDA was examined with SPSS 26.0 (SPSS Inc., Chicago, IL, USA). Chemometric analyses, including principal component analysis (PCA) and orthogonal partial least squares-discriminant analysis (OPLS-DA), were applied using the SIMCA-P Version 14.1 (Umetrics AB, Umea, Sweden).

## 3 Results

### 3.1 C, H, O, and N stable isotopes from different origins

**Figure 4:**
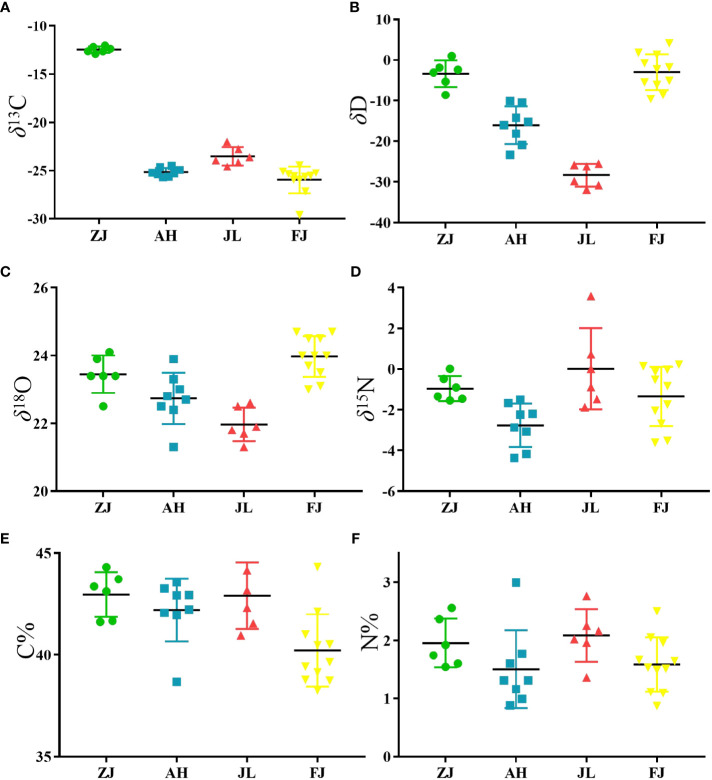
Scatter plot of the variation in **(A)** δ^13^C, **(B)** δD, **(C)** δ^18^O, **(D)** δ^15^N, **(E)** C contents and **(F)** N contents of G.lucidum samples from different origins in China. (ZJ, n=6, AH, n=8, JL, n=6, FJ, n=11).

Six variables, including C, H, O, and N stable isotopes and the C and N contents in *G. lucidum* samples from different provinces (ZJ, AH, JL, and FJ), are compiled in **Table 2**. *G. lucidum* isotope ratios showed *δ* ^13^C values ranging from -29.61 to -12.01, *δ*D values ranging from -31.9 to 4.1, *δ* ^18^O values ranging from 21.3 to 24.7, and *δ* ^15^N values ranging from -4.36 to 3.59. The distribution of four stable isotopes is shown in **Figure 4** to demonstrate the differences among origins.

**Table 2:** Stable isotope values in *G. lucidum* samples from different regions.

Origins	*δ* ^13^C values (‰)	*δ*D values (‰)	*δ* ^18^O values (‰)	*δ* ^15^N values (‰)
ZJ	−12.44 ± 0.33	−3.4 ± 3.3	23.5 ± 0.6	−0.96 ± 0.62
AH	−25.16 ± 0.44	−16.0 ± 4.6	22.7 ± 0.8	−2.76 ± 1.07
JL	−23.51 ± 0.95	−28.3 ± 2.8	22.0 ± 0.5	0.01 ± 2.00
FJ	−25.95 ± 1.38	−3.0 ± 4.4	24.0 ± 0.6	−1.34 ±1.46

### 3.2 Evaluation of chemometric models for *G. lucidum* authentication from different origins

**Figure 5:**
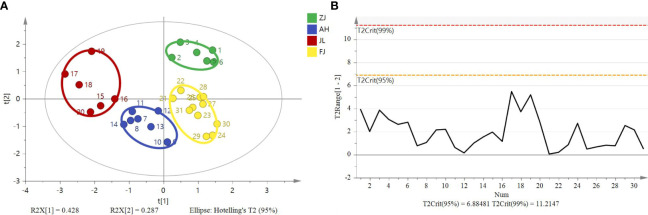
Principal component analysis score plot and Hotelling’s T^2^ ellipse from the isotope ratios (*δ* ^13^C, *δ*D, *δ* ^18^O, and *δ* ^15^N) of *G*. *lucidum* samples from ZJ, AH, JL, and FJ. **(A)** Principal component analysis score plot. **(B)** Hotelling’s T^2^ ellipse was constructed from the PCA score plot. The results are based on the 95% confidence interval.

Using the data of isotope ratios (*δ* ^13^C, *δ*D, *δ* ^18^O, and *δ* ^15^N), an unsupervised multivariate data analysis method, that is, PCA, was performed to minimize dimension and visualize the separation effects of geographical origins. The 2D score plot of the PCA divided the samples into four independent clusters (**Figure 5A**). The results of the classification were consistent with those of the original data. The results are based on the 95% confidence interval (**Figure 5B**).

**Figure 6:**
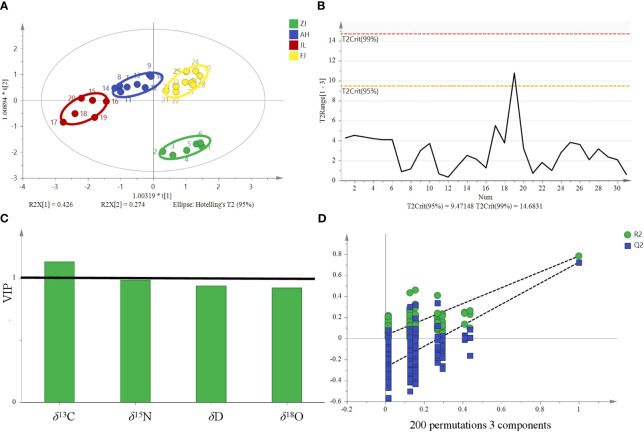
OPLS-DA model based on four stable isotopes (*δ* ^13^C, *δ*D, *δ* ^18^O, and *δ* ^15^N) for tracing the origins of *G*. *lucidum.* **(A)** OPLS-DA score, **(B)** Hotelling’s T^2^ ellipse was constructed from the OPLS-DA score plot. The results are based on the 95% confidence interval. **(C)** VIP plots and **(D)** The permutation test plots of the model for the samples.

OPLS-DA is a modification of the PLS-DA method, where the variation in the data (X matrix) is separated into two parts: one that is linearly related to the response variable (Y matrix) and one that is orthogonal or unrelated. In SIMCA version 14.1 by Umetrics, the OPLS-DA method is implemented to allow multigroup data analysis by performing several binary (one-against-all) comparisons. In the context of a four-group study, four separate OPLS-DA models would be constructed, each focusing on the discrimination of one group against the remaining three. Therefore, these models can then be collectively interpreted to understand the differences among the four groups. OPLS-DA models in SIMCA 14.1 also allow users to visualize and interpret the predictive and orthogonal components separately, which aids in effectively understanding the underlying structure of the dataset. Therefore, OPLS-DA is a supervised discriminant analysis model that can assist in understanding inter-class separation and identifying potential categorical variables, increasing its suitability for multivariate discriminant analysis. The OPLS-DA model was developed utilizing stable isotope ratios as variables to visualize and investigate their intrinsic correlation and explore their contribution to the origin discrimination of *G. lucidum*. The OPLS-DA 2D score plot showed that samples were divided into four categories (**Figure 6A**). The results are based on the 95% confidence interval (**Figure 6B**). The model can explain 91.6% of the variation (*R* ^2^X = 0.916, *R* ^2^Y = 0.726), demonstrating a high predictive capability (*Q* ^2^ = 0.67) and showing a good fit and predictive capability of the training set. Theoretically, in OPLS-DA, VIP values larger than 1 indicate that variables are essential. The VIP (**Figure 6C**) suggested that the *δ* ^13^C value was the most important contributor to the discrimination (VIP > 1). Cross-validation with 200 permutation tests showed that the OPLS-DA model has good robustness (*R^2^ = *0.0295, *Q^2^* = −0.276, **Figure 6D**).

**Figure 7:**
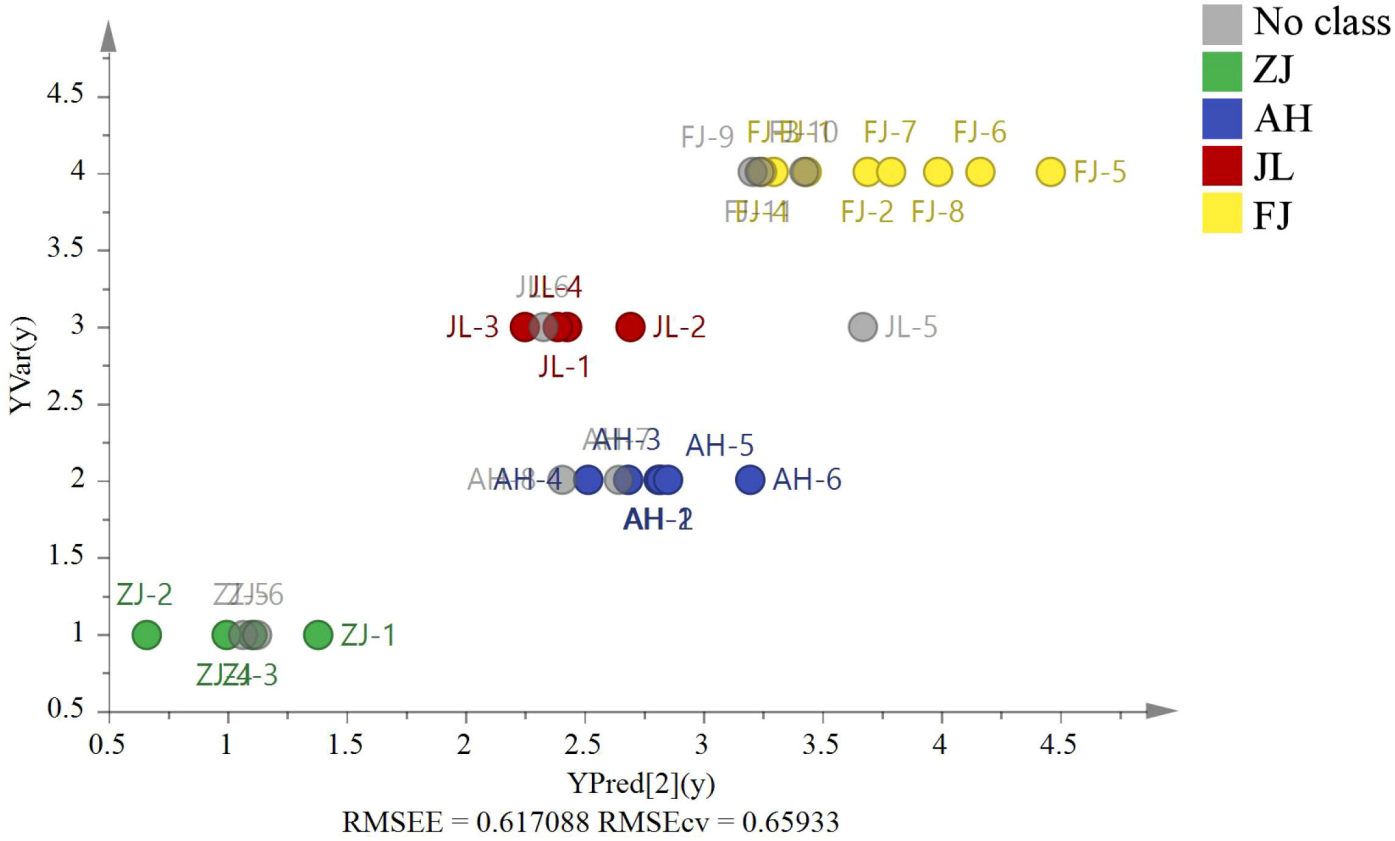
The PLS model based on four stable isotopes (*δ* ^13^C, *δ*D, *δ* ^18^O, and *δ* ^15^N) of origin validation by nine external *G. lucidum* samples (ZJ=2, AH=2, JL=2, and FJ=3).

*G. lucidum* samples (ZJ = 2, AH = 2, JL = 2, and FJ = 3) were used for external source validation. **Figure 7** shows the predicted and actual values of the PLS validation according to the isotope ratios (*RMSEE* = 0.62, *RMSECV* = 0.66). The results revealed that the model could identify nine external samples in their classified group with 100% accuracy.

**Figure 8:**
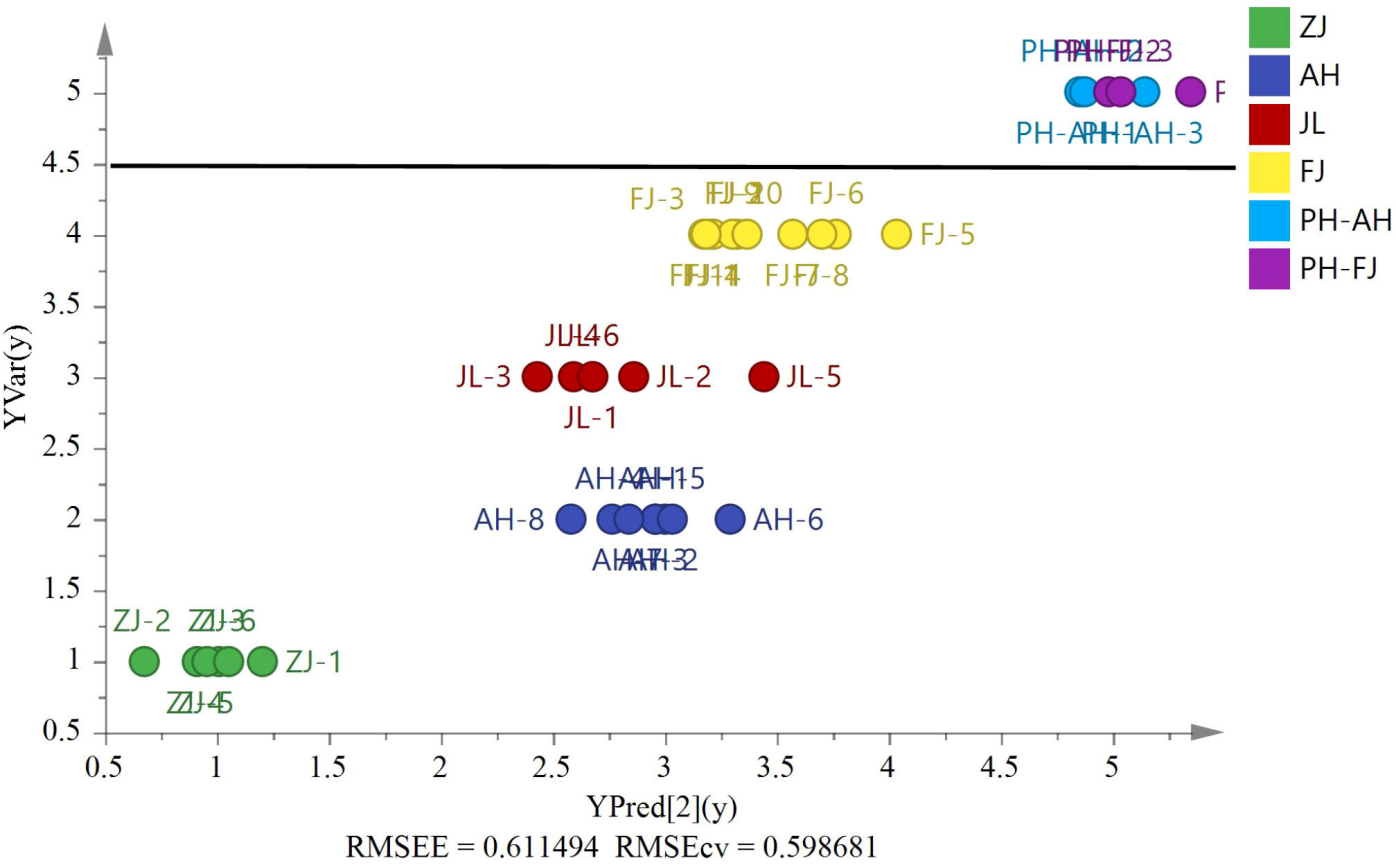
The PLS model based on four stable isotopes (*δ* ^13^C, *δ*D, *δ* ^18^O, and *δ* ^15^N) of origin validation by the heterogeneous six *Pseudostellariae Radix* samples (AH, *n*=3 and FJ, *n*=3).

Six *Pseudostellaria heterophylla* heterogeneous samples were incorporated at random, including three samples from Fujian and others from Anhui, to further confirm the reliability of the model. *δ* ^13^C, *δ*D, *δ* ^18^O, and *δ* ^15^N were acquired by using the same isotope ratio mass spectrometry for verification of other heterogeneous samples (**Figure 8**, *RMSEE* = 0.61, *RMSECV* = 0.60). **Figure 8** shows the classification of *G. lucidum* and *Pseudostellaria heterophylla* samples with a cutoff value of 4.5. The result indicated that *G. lucidum* has its own unique stable isotopes. Moreover, the results revealed that the model could identify other heterogeneous samples (Pseudostellariae Radix) in other groups with 100% accuracy. By contrast, these samples (*G. lucidum* and *Pseudostellaria heterophylla*) were again analyzed by PCA (**Figure S1**). The analysis results showed that *G. lucidum* was clearly isolated from samples of *Pseudostellaria heterophylla*. Therefore, *G. lucidum* is efficiently and precisely identified using the established model.

FLDA was employed to establish a predictive model to facilitate the clustering of group members based on observed characteristics. The discriminant function based on linear combinations of predictor variables was utilized to discriminate further and classify the unknown members. The FLDA model of the geographical origin of *G. lucidum* was established, and the classification results are shown in **Table 3**. The FLDA function is as follows: *Y*(ZJ) = −18.131 *δ* ^13^C + 3.131 *δ* ^15^N + 70.205 *δ* ^18^O − 0.316 *δ*D − 932.103, *Y*(AH) = −47.559 *δ* ^13^C − 3.887 *δ* ^15^N + 62.028 *δ* ^18^O − 6.324 *δ*D − 1371.246, *Y*(JL) = −46.931 *δ* ^13^C − 2.976 *δ* ^15^N + 57.780 *δ* ^18^O − 7.040 *δ*D − 1275.817, and *Y*(FJ) = −43.875 *δ* ^13^C − 1.462 *δ* ^15^N + 67.994 *δ* ^18^O − 4.505 *δ*D − 1394.239. The FLDA linear discriminant with stable isotopes as discriminant variables has been evaluated with back-replacement accuracy of 100% and cross-validation reaching 95.7%.

**Table 3:** The discrimination model based on four stable isotopes (*δ* ^13^C, *δ*D, *δ* ^18^O, and *δ* ^15^N) of the geographical origin of *G. lucidum*.

	Origin	Predicted group membership				Total
		ZJ	AH	JL	FJ	
Geographical origin	ZJ	4	0	0	0	4
	AH	0	6	0	0	6
	JL	0	0	4	0	4
	FJ	0	0	0	9	9
Cross-validated	ZJ	4	0	0	0	4
	AH	0	6	0	0	6
	JL	0	0	4	0	4
	FJ	0	1	0	8	9
External-validated	ZJ	2	0	0	0	2
	AH	0	2	0	0	2
	JL	0	0	2	0	2
	FJ	0	0	0	2	2

**Figure 9:**
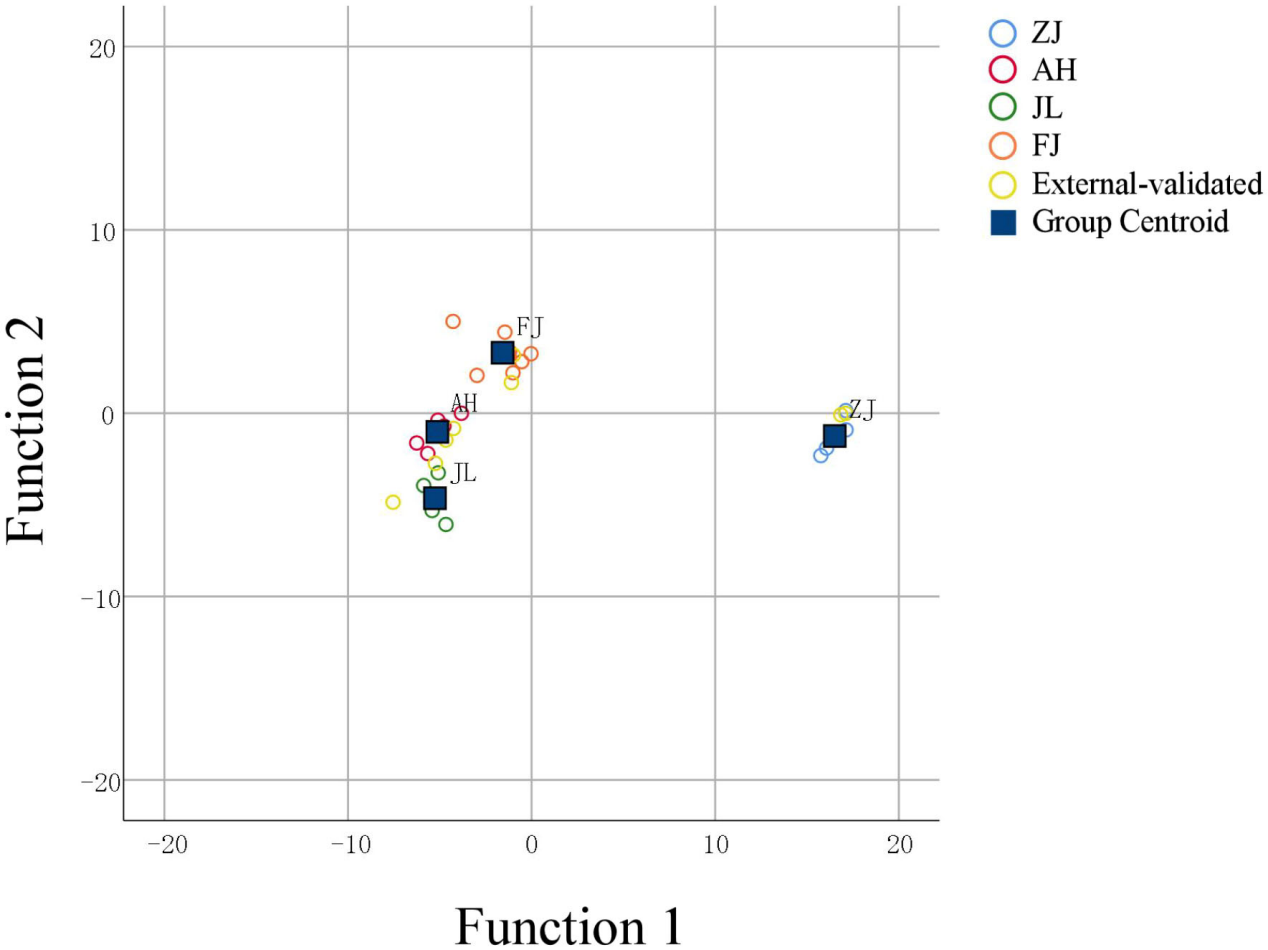
FLDA of four stable isotopes (*δ* ^13^C, *δ*D, *δ* ^18^O, and *δ* ^15^N) for *G.lucidum*.

Scatter plots were used to evaluate the distribution of the geographical origins to distinguish the discrepancies explicitly. As shown in **Figure 9**, geographical origins could be successfully divided into four groups. The discriminant function is based on linear combinations of predictor variables to discriminate further and classify the unknown members. The model for geographical origin discrimination was used to verify the origins of nine samples outside the model. The nine *G. lucidum* samples were accurately recognized, demonstrating the precision and consistency of the model.

### 3.3 C, H, O, and N stable isotopes from different stages

**Figure 10:**
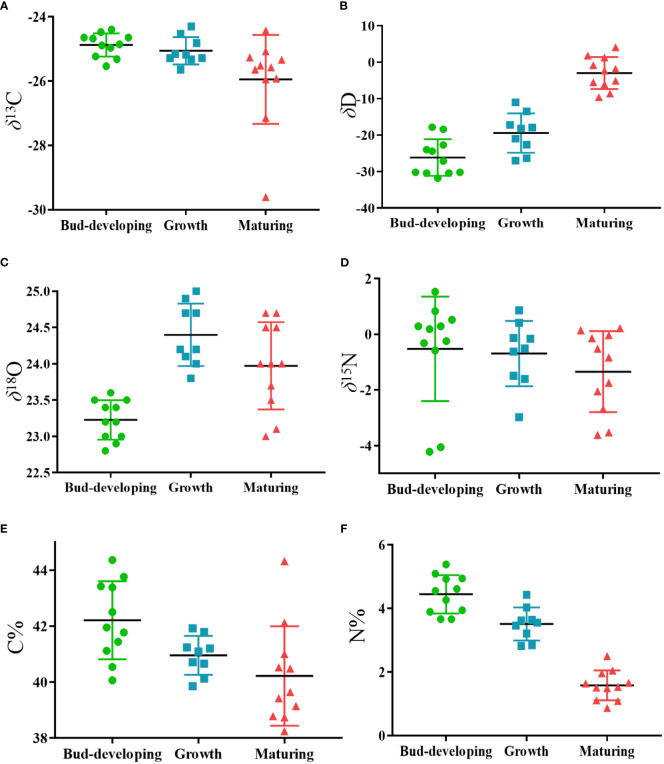
Scatter plot of the variation in **(A)** δ^13^C, **(B)** δD, **(C)** δ^18^O, **(D)** δ^15^N, **(E)** C contents and **(F)** N contents of G.lucidum samples at different stages. (Bud-developing stage, *n*=11, Growth stage, *n*=9, Growth stage, *n*=11).

Thus far, studies that have exhaustively examined the relationship between stable isotopes and geographical origins at various phases are currently unavailable. Four stable isotopes and C and N contents are shown in **Table 4** and **Figure 10**, respectively. *G. lucidum* stable isotope showed *δ* ^13^C values ranging from −29.61 to −24.30, *δ*D values ranging from −31.8 to 4.1, *δ* ^18^O values ranging from 22.8 to 25.0, and *δ* ^15^N values ranging from −4.22 to 1.53. The average *δ*D values of samples at the maturing stage were highest (−3.0‰), followed by the growth and bud development stages.

**Table 4:** Stable isotope values in *G. lucidum* samples at different stages.

Growth stages	*δ* ^13^C values (‰)	*δ*D values (‰)	*δ* ^18^O values (‰)	*δ* ^15^N values (‰)
Bud-developing	−24.88 ± 0.36	−26.1 ± 5.0	23.2 ± 0.3	−0.52 ± 1.88
Growth stage	−25.06 ± 0.43	−19.4 ± 5.4	24.4 ± 0.4	−0.69 ± 1.17
Maturing stage	−25.95 ± 1.38	−3.0 ± 4.4	24.0 ± 0.6	−1.34 ±1.46

### 3.4 Correlation analysis of *δ* ^13^C and *δ* ^15^N values with C and N contents from different stages

The correlation between carbon and nitrogen can reflect the nutrient acquisition of *G. lucidum* during the growth process, which plays an important role in its growth (Hsieh and Yang, 2004). Correlations of *δ* ^13^C and *δ* ^15^N values with C and N contents in *G. lucidum* are shown in **Table 5**. A significantly negative relationship is observed between the C/N ratio and the N content (*r* = −0.894, *p* < 0.001). The relationship between the carbon-to-nitrogen ratio and nitrogen content is one of mutual inhibition.

**Table 5:** Correlation analysis table of *δ* ^13^C, *δ* ^15^N, and C, N contents stoichiometry in *G. lucidum* at different stages.

Correlation coefficient	*δ* ^13^C	*δ* ^15^N	C%	N%	C/N ratio
*δ* ^13^C	1	0.691	0.206	0.001	0.005
*δ* ^15^N	−0.074	1	0.638	0.164	0.133
C%	0.234	−0.088	1	0.022	0.271
N%	0.553*	0.256	0.410*	1	0.000
C/N ratio	−0.491*	−0.276	−0.204	−0.894***	1

The lower left corner is the correlation coefficient and the upper right corner is the significant level.

* means significant difference, P<0.05, *** means extremely significant difference, P<0.001.

### 3.5 Evaluation of chemometric models for *G. lucidum* authentication from different stages

**Figure 11:**
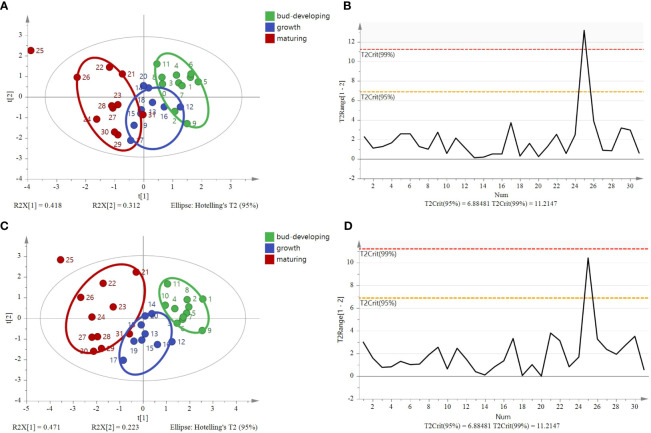
Principal component analysis (PCA) and Hotelling’s T^2^ ellipse of *G*. *lucidum* samples at stages of the bud-developing stage, growth stage, and maturing stage. **(A)** Principal component analysis score plot of four variables (*δ* ^13^C, *δ*D, *δ* ^18^O, *δ* ^15^N), **(B)** Hotelling’s T^2^ ellipse of four variables was constructed from the PCA score plot. The results are based on the 95% confidence interval. **(C)** Principal component analysis score plot of six variables (*δ* ^13^C, *δ*D, *δ* ^18^O, *δ* ^15^N C%, and N%) and **(D)** Hotelling’s T^2^ ellipse of six variables constructed from the PCA score plot. The results are based on the 95% confidence interval.

**Figure 12:**
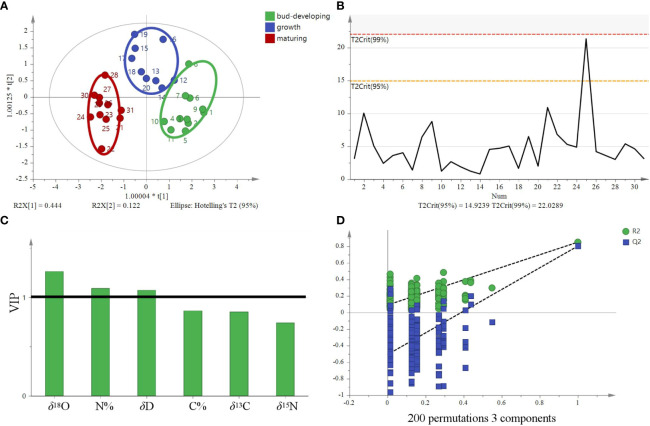
OPLS-DA model based on six variables (*δ* ^13^C, *δ*D, *δ* ^18^O, *δ* ^15^N, C%, and N%) for tracing the different stages of *G*. *lucidum.* **(A)** OPLS-DA score, **(B)** Hotelling’s T^2^ ellipse constructed from the OPLS-DA score plot. The results are based on the 95% confidence interval. **(C)** VIP plots and **(D)** The permutation test plots of the model for the samples.

The model based on stable isotope ratios fails to distinguish samples at different stages and overlaps in some areas (**Figure 11A**). Compared with the model of stable isotopes (four variables, including *δ* ^13^C, *δ* ^15^N, *δ*D, and *δ* ^18^O), the PCA model, after increasing C% and N%, showed that *G. lucidum* samples at each stage were highly centralized (**Figure 11C**). Different stages were clustered on the left of PC1. The results are based on the 95% confidence interval (**Figures 11B, D**). The OPLS-DA score plot showed that the samples were divided into three categories, with significant differences (**Figure 12A**). The model could explain 99.0% of the variation (*R^2^X* = 0.991, *R^2^Y* = 0.74) and had high prediction capability (*Q^2^ = *0.638). The results are based on the 95% confidence interval (**Figure 12B**). The VIP of six variables showed that *δ* ^18^O, N%, and *δ* ^15^N are the most important contributors to the discrimination (VIP > 1, **Figure 12C**). Cross-validation with 200 permutation tests showed that the OPLS-DA model has good robustness (*R^2^ = *0.0915 and *Q^2^* = −0.508, **Figure 12D**).

**Figure 13:**
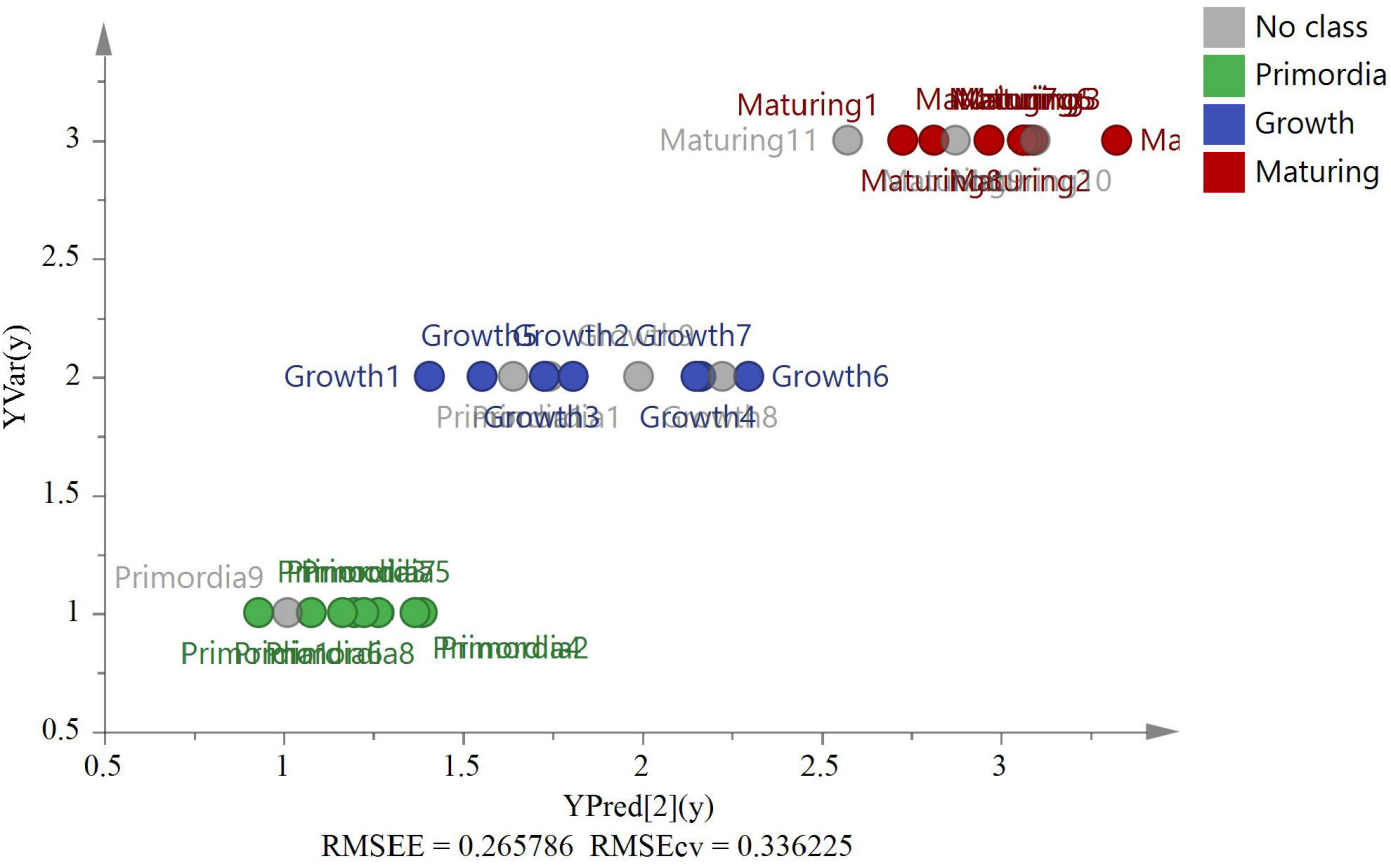
The PLS model based on six variables (*δ* ^13^C, *δ*D, *δ* ^18^O, *δ* ^15^N, C%, and N%) of stages validation by eight external *G. lucidum* samples (Bud-developing stage *n*=3, Growth stage *n*=2, and Maturing stage *n*=3).

Growth stage samples were used for the model verification. **Figure 13** shows the predicted and actual values of the PLS model according to the four isotope ratios (*δ* ^13^C, *δ*D, *δ* ^18^O, and *δ* ^15^N) and C% and N% (*RMSEE* = 0.27, *RMSECV* = 0.34). All external sample validations of *G. lucidum* were classified correctly.

The eigenvalues of the sample correlation matrix were calculated, and the eigenvalue demonstrates the importance of each new factor in explaining data variability. The contribution of six variables, namely, *δ* ^13^C, *δ*D, *δ* ^18^O, *δ* ^15^N, C%, and N%, was analyzed by PCA at the PCA model, and the results are presented in **Table 6**. Three major factors generally accounted for 87.578% (factors 1, 2, and 3 explained 47.078%, 22.266%, and 18.234% of the data variability, respectively) of the six variations inherent in the input model data. Among them, N% was strongly associated with factor 1 (0.878), and C% was highly correlated with factor 2 (−0.854), indicating that the contribution rates of C% and N% were highly correlated with the first two factors, thereby boosting the model’s accuracy, especially N% (see **Table 7**). The contribution of six variables was also analyzed using the VIP factor in the OPLS-DA model, and the results are presented in **Figure 12C**. *δ*D, N%, and *δ* ^15^N VIP values >1 indicate that these markers contribute to differentiation at different stages. This finding also showed the importance of N% in the model.

**Table 6:** Three exploratory Factor Analysis: Eigenvalues of three factors.

Factor	Eigenvalue	(%) Total Variance	Cumulative (%)
1	2.825	47.078	47.078
2	1.336	22.266	69.344
3	1.094	18.234	87.578

**Table 7:** First exploratory Factor Analysis: Relationship between response variables and factors.

Variables	Factor 1	Factor 2	Factor 3
*δ* ^13^C	0.848	0.078	−0.193
*δ*D	−0.918	0.250	−0.082
*δ* ^18^O	−0.007	0.902	−0.240
*δ* ^15^N	0.048	−0.030	0.970
C%	0.292	−0.854	−0.205
N%	0.878	−0.264	0.263

## 4 Discussion

*G. lucidum* is a popular medicinal fungus that has been used in traditional medicine for decades, with its origin influencing its medicinal and commercial worth. The amount of active ingredients and the price of *G. lucidum* from different origins varies significantly; hence, fraudulent labeling is common. Reliable techniques for *G. lucidum* geographic verification are urgently required to safeguard the interests of consumers, producers, and honest dealers. A stable isotope is widely acknowledged as a useful traceability technique and could be developed to confirm the geographical origin of *G. lucidum*. The chemical composition of *G. lucidum* was affected by different regions and planting stages caused by temperature, humidity, CO_2_ content, and other environmental factors (Sudheer et al., 2018a; Sudheer et al., 2018b). Particularly during the planting process of *G. lucidum*, temperature, humidity, and CO_2_ content directly affect its growth states (bud development, growth, and maturing stages), followed by changes in *G. lucidum* stable isotopes along with bioactive compounds in these stages. The changes in bioactive compounds may contribute to the variations of stable isotopes at different stages. Therefore, the variation in stable isotopes in different regions and stages should be comprehensively considered.

The latitude is one of the main factors affecting the *δ* ^13^C values; theoretically, high *δ* ^13^C values will have high latitudes (Guo et al., 2017; Li et al., 2011). The latitudes of the four provinces are ranked as follows: Jilin > Anhui > Zhejiang > Fujian. Thus, the theoretical arrangement of *δ* ^13^C values should follow the order: Jilin > Anhui > Zhejiang > Fujian. However, in the actual data, the Zhejiang samples with *δ* ^13^C values were the highest, and those of the other producing regions were within acceptable bounds. Moreover, *δ* ^13^C values had minimal effect on the separation of *G. lucidum* at different growth stages (VIP < 1). In the origin model, *δ* ^13^C values showed the strongest influence on the separation of different regions (**Figure 6A**). The significance of this effect was also observed in **Figure 6C** (VIP > 1). Thus, *δ* ^13^C values were an important variable in *G. lucidum* from different origins. Six other heterogeneous samples (*Pseudostellaria heterophylla)* of the same origin, including three from Fujian (FJ) and three from Anhui (AH), were randomly introduced to further verify the reliability of the model. *δ* ^13^C, *δ*D, *δ* ^18^O, and *δ* ^15^N were acquired by using the same isotope ratio mass spectrometry. The result of *G. lucidum* PCA, OPLS-DA, and PLS analysis to further verify the reliability of the model showed that other samples (*Pseudostellaria heterophylla*) could be identified by the model in another group with 100% accuracy. These samples (*G. lucidum* and *Pseudostellaria heterophylla*) were then again analyzed by PCA, which revealed that *G. lucidum* samples were separated from *Pseudostellaria heterophylla* samples. These results demonstrated that *G. lucidum* had its own unique stable isotopes, while other TCM from the same location and the known model could be used to distinguish *G. lucidum* accurately and effectively. For the model validation, nine of the *G. lucidum* as external samples for the OPLS-DA model showed that external samples could be identified by the model in their group with 100% accuracy. In the FLDA method, the eight *G. lucidum* samples consistent with PLS for discrimination and classification results were the same at 100% accuracy, indicating the accuracy and stability of the established model.

Studies have shown that the contents of different substances vary significantly in different stages. The highest content of ganoderic acids A, B, D, and F was detected during the bud development stage. The highest contents of ganoderiol and ganoderic acids S and T were detected during the maturing stage (Zhou et al., 2018). These components are not only related to growing conditions (i.e., latitude, longitude, temperature, and altitude) but also have different growth stages. However, methods for distinguishing between the various growth stages of *G. lucidum* are currently unavailable. Samples of *G. lucidum* at various stages were collected in Fujian for this experiment to further examine the formation mechanism of stable isotopes. Focusing only on the maturing stage, the stable isotope ratio of *G. lucidum* was stable (RSD < 4.4%). The ANOVA results showed differences in *δ* ^18^O (*p* < 0.05), N% (*p* < 0.001), and *δ*D (*p* < 0.001), indicating that *δ*D, N%, and *δ* ^18^O values can be used to distinguish *G. lucidum* at different stages. C% and N% showed the maximum concentrations at the bud development stage. These concentrations declined with the growth of *G. lucidum*, which may be associated with the maturation stage. The discriminative stage model of *G. lucidum* had six variables (*δ* ^13^C, *δ*D, *δ* ^18^O, *δ* ^15^N, C%, and N%) whereas the origin model had four variables (*δ* ^13^C, *δ*D, *δ* ^18^O, and *δ* ^15^N). Evidently, PCA results demonstrated an overlapping phenomenon when only four variables were employed to separate samples at different stages, and miscalculation was observed when PLS was used to validate the model at the overlapped sample (e.g., S12, S15, S17, and S19). The patterns of *G. lucidum* samples at each stage were distinguishable following the addition of the C% and N% variables, and the model validation of the PLS demonstrated that the accuracy had reached 100%. Further study of the contribution of six variables using principal component factor analysis in the PCA model revealed that the contribution rates of C% and N% in the phase model could not be disregarded, especially N%. Similar to PCA and the contribution of six variables by the VIP factor in the OPLS-DA model, N% was the major VIP marker at different stages. Therefore, the stage model with six variables was superior to the model with only four variables. This finding may be due to the same geographical location of samples collected at successive phases, which was difficult to identify with only four variables (*δ* ^13^C, *δ*D, *δ* ^18^O, and *δ* ^15^N). After increasing the C% and N% with stage characteristics, the model could be successfully applied to the differentiation of stages and accurate validation. By contrast, the six variables (*δ* ^13^C, *δ*D, *δ* ^18^O, *δ* ^15^N, C%, and N%) in different stages of the current study may also provide a reference for the cultivation of *G. lucidum.* These variables could not only be used to trace geographical origin but also for tracking the migration of the flux of nutritional elements such as N during cultivation (Tan et al., 2019).

## 5 Conclusions

This study determined the C, H, O, and N stable isotopes in *G. lucidum* samples from different regions. The data were evaluated using chemometric analyses, including PCA, OPLS-DA, PLS, and FLDA. These results suggested that the geographic origin significantly impacted these *G. lucidum* indicators. The findings of this study could be used to boost the transparency of *G. lucidum* supply chains. In the future, it will be necessary to collect additional samples from different years and integrate mean temperature, precipitation, and geographic information systems to develop a comprehensive chemometric prediction model that comprehends the effect of the geographical environment or climate and serves as a standard for quality control.

## Data availability statement

The original contributions presented in the study are included in the **Supplementary Material**, and further inquiries can be directed to the corresponding authors.

## Author contributions

YZ conducted experiments and data analysis. CW and KJ provided and analyzed sample sources. WenX and MY contributed to the conception and design of the study. SC, LW, XZ, WeiX, and YL designed, supervised the work, and prepared the manuscript. All authors contributed to the manuscript revision, read, and approved the submitted version.
