# Supplementary material for: Quality control of Ganoderma lucidum by using C, H, O, and N stable isotopes and C and N contents for geographical traceability
Source: Front Plant Sci. 2023 Oct 11;14:1234729. doi: 10.3389/fpls.2023.1234729 (PMC10598867; doi:10.3389/fpls.2023.1234729)
Supplement: Supplementary file 1 [file DataSheet_1.docx]

**Supplementary Material**

**Supplementary Figure 1.** Principal component analysis score plot from the isotope ratios (*δ*^13^C, *δ*D, *δ*^18^O, *δ*^15^N ) of *G. lucidum* samples (ZJ=2, AH=2, JL=2, FJ=3) and *Pseudostellaria heterophylla* samples (AH=3, FJ=3)

**Supplementary Table1.** Raw data of four stable isotopes, C% and N% from different regions.

| NO. | *δ*13CVPDB ‰ | *δ*DV-SMOW‰ | *δ*18OV-SMOW‰ | *δ*15NAir ‰ | CTOC (%) | NTON (%） |
| --- | --- | --- | --- | --- | --- | --- |
| S1 | -12.61 | -2.4 | 24.1 | -0.48 | 43.72 | 2.56 |
| S2 | -12.36 | -5.3 | 22.5 | -1.35 | 44.31 | 1.54 |
| S3 | -12.01 | -8.6 | 23.4 | 0.01 | 41.66 | 2.36 |
| S4 | -12.16 | -3.1 | 23.4 | -0.90 | 41.61 | 1.60 |
| S5 | -12.89 | 1.0 | 23.4 | -1.46 | 43.36 | 1.74 |
| S6 | -12.62 | -1.9 | 23.9 | -1.55 | 43.12 | 1.92 |
| S7 | -25.37 | -16.0 | 22.5 | -2.20 | 41.95 | 1.60 |
| S8 | -25.70 | -18.1 | 22.4 | -2.24 | 42.23 | 1.31 |
| S9 | -25.29 | -15.2 | 23.3 | -4.17 | 38.68 | 0.99 |
| S10 | -25.21 | -10.1 | 22.8 | -4.36 | 43.26 | 1.16 |
| S11 | -24.49 | -23.3 | 23.0 | -1.67 | 43.57 | 2.99 |
| S12 | -24.63 | -20.9 | 23.9 | -1.51 | 42.06 | 1.77 |
| S13 | -24.93 | -14.2 | 22.7 | -3.07 | 42.94 | 1.31 |
| S14 | -25.64 | -10.5 | 21.3 | -2.87 | 42.92 | 0.88 |
| S15 | -23.92 | -25.5 | 21.9 | -1.48 | 45.31 | 2.16 |
| S16 | -23.61 | -25.8 | 22.6 | -0.88 | 44.16 | 2.76 |
| S17 | -22.02 | -31.9 | 21.3 | 0.72 | 43.17 | 2.25 |
| S18 | -22.76 | -30.8 | 21.7 | 0.01 | 42.32 | 1.96 |
| S19 | -24.59 | -26.2 | 22.5 | 3.59 | 41.54 | 2.02 |
| S20 | -24.15 | -29.8 | 21.8 | -1.88 | 40.95 | 1.36 |
| S21 | -25.64 | -2.3 | 23.0 | -0.52 | 44.32 | 1.53 |
| S22 | -25.95 | 1.8 | 23.1 | 0.22 | 40.52 | 1.09 |
| S23 | -25.26 | -1.8 | 23.7 | -2.05 | 42.12 | 1.11 |
| S24 | -25.92 | -0.8 | 24.0 | -3.53 | 41.00 | 0.87 |
| S25 | -29.61 | 4.1 | 23.5 | -0.03 | 39.64 | 1.49 |
| S26 | -27.14 | 1.3 | 24.0 | 0.14 | 39.42 | 1.51 |
| S27 | -25.52 | -5.5 | 24.5 | -0.83 | 38.24 | 1.66 |
| S28 | -25.57 | -6.1 | 24.7 | -0.14 | 38.74 | 2.05 |
| S29 | -25.34 | -9.6 | 24.5 | -3.62 | 39.14 | 1.96 |
| S30 | -25.07 | -5.1 | 24.7 | -2.68 | 38.78 | 1.64 |
| S31 | -24.43 | -8.6 | 24.0 | -1.75 | 40.48 | 2.50 |

**Supplementary Table2.** Raw data of four stable isotopes, C% and N% content at different stages.

| No. | *δ*13CVPDB ‰ | *δ*DV-SMOW‰ | *δ*18OV-SMOW‰ | *δ*15NAir ‰ | CTOC (%) | NTON (%） |
| --- | --- | --- | --- | --- | --- | --- |
| S1 | -24.86 | -30.2 | 22.9 | -0.32 | 43.38 | 4.92 |
| S2 | -25.23 | -30.4 | 23.0 | -4.22 | 44.36 | 3.89 |
| S3 | -24.65 | -24.4 | 23.4 | 0.29 | 40.06 | 5.09 |
| S4 | -24.89 | -24.0 | 23.2 | 0.52 | 41.12 | 4.27 |
| S5 | -24.40 | -30.4 | 23.0 | -0.24 | 41.44 | 3.94 |
| S6 | -24.65 | -30.2 | 23.5 | 1.53 | 40.54 | 4.61 |
| S7 | -24.68 | -27.1 | 23.5 | 0.19 | 41.77 | 4.55 |
| S8 | -25.32 | -22.7 | 23.6 | 0.83 | 43.76 | 4.94 |
| S9 | -24.48 | -31.8 | 23.4 | -4.06 | 43.42 | 5.38 |
| S10 | -24.97 | -18.4 | 23.2 | -0.58 | 41.95 | 3.66 |
| S11 | -25.54 | -17.8 | 22.8 | 0.29 | 42.50 | 3.66 |
| S12 | -24.30 | -27.0 | 24.1 | -0.51 | 40.71 | 4.43 |
| S13 | -25.28 | -22.6 | 24.2 | -0.62 | 40.12 | 3.55 |
| S14 | -25.64 | -21.0 | 23.8 | -0.16 | 41.20 | 3.64 |
| S15 | -24.53 | -11.0 | 24.7 | -0.13 | 41.24 | 3.62 |
| S16 | -24.82 | -26.3 | 24.7 | 0.41 | 40.66 | 4.03 |
| S17 | -25.18 | -17.2 | 25.0 | -2.98 | 39.85 | 3.21 |
| S18 | -25.33 | -17.9 | 24.2 | -1.49 | 41.92 | 2.82 |
| S19 | -25.29 | -18.2 | 24.9 | -1.61 | 41.79 | 2.84 |
| S20 | -25.16 | -13.5 | 24.0 | 0.86 | 41.09 | 3.46 |
| S21 | -25.64 | -2.3 | 23.0 | -0.52 | 44.32 | 1.53 |
| S22 | -25.95 | 1.8 | 23.1 | 0.22 | 40.52 | 1.09 |
| S23 | -25.26 | -1.8 | 23.7 | -2.05 | 42.12 | 1.11 |
| S24 | -25.92 | -0.8 | 24.0 | -3.53 | 41.00 | 0.87 |
| S25 | -29.61 | 4.1 | 23.5 | -0.03 | 39.64 | 1.49 |
| S26 | -27.14 | 1.3 | 24.0 | 0.14 | 39.42 | 1.51 |
| S27 | -25.52 | -5.5 | 24.5 | -0.83 | 38.24 | 1.66 |
| S28 | -25.57 | -6.1 | 24.7 | -0.14 | 38.74 | 2.05 |
| S29 | -25.34 | -9.6 | 24.5 | -3.62 | 39.14 | 1.96 |
| S30 | -25.07 | -5.1 | 24.7 | -2.68 | 38.78 | 1.64 |
| S31 | -24.43 | -8.6 | 24.0 | -1.75 | 40.48 | 2.50 |

S21-S31 in Table1 and Table2 are the same batch of samples.

**Supplementary Table 3.** Raw data of four stable isotopes of *Pseudostellariae heterophylla*.

| No. | *δ*13CVPDB ‰ | *δ*DV-SMOW‰ | *δ*18OV-SMOW‰ | *δ*15NAir ‰ |
| --- | --- | --- | --- | --- |
| S1 | -27.31 | -82.4 | 25.5 | 1.07 |
| S2 | -26.95 | -78.7 | 26.1 | 0.59 |
| S3 | -27.14 | -79.4 | 26.1 | 3.09 |
| S4 | -25.79 | -67.8 | 27.9 | 3.57 |
| S5 | -26.07 | -67.7 | 27.0 | 1.72 |
| S6 | -25.71 | -71.0 | 27.4 | 1.62 |
